# Theoretical Prediction of Ultrasound Elastography for Detection of Early Osteoarthritis

**DOI:** 10.1155/2013/565717

**Published:** 2013-11-05

**Authors:** Lan Wang, Shigao Chen, Kai-Nan An, Hui-Lin Yang, Zong-Ping Luo

**Affiliations:** ^1^Orthopaedic Institute, The 1st Affiliated Hospital Soochow University, 708 Renmin Road, Suzhou, Jiangsu 215007, China; ^2^Department of Orthopedics of the 1st Affiliated Hospital Soochow University, 708 Renmin Road, Suzhou, Jiangsu 215007, China; ^3^Basic Ultrasound Research Laboratory, Department of Physiology and Biomedical Engineering, Mayo Clinic College of Medicine, Rochester, MN 55905, USA; ^4^Biomechanics Laboratory, Division of Orthopedic Research, Mayo Clinic College of Medicine, Rochester, MN 55905, USA

## Abstract

Ultrasound elastography could be used as a new noninvasive technique for detecting early osteoarthritis. As the first critical step, this study theoretically predicted the excitation power and the measurement errors in detecting cartilage detect. A finite element model was used to simulate wave propagation of elastography in the cartilage. The wave was produced by a force *F*, and the wave speed *C* was calculated. The normal cartilage model was used to define the relationship between the wave speed and elastic modulus. Various stiffness values were simulated. *F* = 10 N with a duration of 0.5 ms was required for having measurable deformation (10 *μ*m) at the distal site. The deformation had a significant rise when the wave crossed the defect. The relationship between the wave speed and elastic parameters was found as *C* = 1.57 × (*E*)/(2 × *ρ(*1+*μ*)))^1/2^, where *E* was the elastic modulus, *μ* was Poisson's ratio, and *ρ* was the density. For the simulated defect with an elastic modulus of 7 MPa which was slightly stiffer than the normal cartilage, the measurement error was 0.1 MPa. The results suggested that, given the simulated conditions, this new technique could be used to detect the defect in early osteoarthritis.

## 1. Introduction

 The early diagnosis is a critical component in the treatment and prevention of osteoarthritis (OA) [1–3]. Currently, research of early OA detection focuses largely on measuring structural changes using techniques of radiography [4], scintigraphy [5], dual-energy X-ray absorptiometry [6], arthroscopy [7], and magnetic resonance imaging (MRI) [8], or on using biomarkers through biological specificity [9, 10]. However, studies showed that the incubation period of OA after cartilage injuries might be up to 2–5 years, and only 20–50% patients had trauma symptoms and movement disorder, suggesting that early symptomatic and structural changes were minimal [11]. The limited structural changes pose challenges in the structural-based diagnostic methods. Meanwhile, biomarker techniques also face challenges from uncertainty in locking one or several markers from a considerable number of inflammatory cytokines for the early defect [12].

As a noninvasive method of quantifying mechanical properties of soft tissues, elastography has been successfully used in detecting lesions and pathological changes of various tissues or organs, including skeletal muscle, cardiac muscle, liver, prostate, breast, and thyroid [13–16]. By using either ultrasound or MRI, elastography detected the propagation of shear wave passing through the tested areas and calculated elastic modulus changes in the tested area from the shear wave propagation speed [17–19]. Several theoretical models were proposed for determining and calculating the elastic modulus changes [19–21].

In contrast to limited macrostructural changes in early cartilage degeneration, early component changes are substantial [22–24]. The normal cartilage consists of more than 90% type II collagen, while degeneration causes significant reduction of type II collagen and increase of type I collagen. Mechanically, type I collagen can be up to 73 times stiffer than type II collagen (366 versus 5 MPa in the elastic modulus) [25]. Therefore, it was much more sensitive to detect subtle changes in the cartilage by the use of mechanical stiffness rather than structural parameters.

The current elastography, however, cannot be directly applied to the cartilage due to the following facts. (1) The cartilage is much stiffer than those soft tissues to which the technique has been used successfully (e.g., 2–70 KPa in the liver versus 5 MPa in the cartilage in the elastic modulus). The stiffer structure leads to rapid energy attenuation and an insufficient excitation power to produce measurable deformation. Simple increase of the excitation power may exceed the predefined safety threshold causing tissue damage [20, 26]. (2) Elastography requires a theoretical model to calculate the elastic modulus from the measured local deformation generated by the shear wave propagation. Since the cartilage is a thin layer structure, the propagation in cartilage is much more complicated than that in the tissues studied to date in which the propagation medium is assumed to be infinite in comparison to the shear wave wavelength. This geometrical restriction leaves no valid theoretical model for determination of the cartilage elastic modulus.

As a first approach to apply the elastography to the diagnosis of early OA, this study developed a theoretical framework to simulate ultrasound shear wave propagation in the cartilage, to quantify the shear wave dispersion, to define new measurement scheme in the excitation power, and to determine relationship between the elastic modulus and shear wave propagation. The model was then used to simulate early defect of the cartilage and to define the minimal detectable defect.

## 2. Methods

### 2.1. Theoretical Model of Cartilage

 A cartilage layer was simulated by a finite element model of 100 mm in length, 100 mm in width, and 5 mm in thickness, a simplified dimension of a typical adult human tibial plateau [27, 28]. The material properties of the normal cartilage simulated included the elastic modulus *E* (5 MPa), Poisson's ratio *μ* (0.3), and the material density *ρ* (1.0 × 10^3^ kg/m^3^) [25, 29]. Three-dimensional linear eight-node elements were used uniformly throughout the model [30, 31]. The size of element was 1 × 1 × 2.5 mm^3^. Lower surface of the model was fixed to a rigid surface simulating the subchondral bone. The transient dynamic analysis was used to quantify the shear wave propagation up to 2 ms when the shear wave reached the distal site of the cartilage and the time step was 10^−3^ ms.

### 2.2. Determination of the Excitation Power

 Clinically, the ultrasound excitation source had to be placed noninvasively on the skin near the cartilage. In this simulation, the excitation source was located at the middle point of one edge. The excitation was a pulse pushing force satisfying two criteria. (1) At the proximal site where the excitation power was the highest, the maximal shear deformation should not cause any damage of the cartilage. (2) At the distal site where the shear wave was attenuated, the shear deformation had to be detected by the ultrasound sensor. Because of the linearity, a unit pulse pushing force (*F* = 1 N) was applied and the shear deformation was calculated. The pulse pushing force magnitude was then determined by the unit pushing force multiplying a factor which was obtained after meeting the given deformation criteria at the proximal site or the distal site whichever came first. The maximal allowed shear deformation at the proximal site was defined as 2 mm in [32], and the minimal shear deformation at the distal site was defined as 10 *μ*m which was the resolution of the elastography currently used [33].

### 2.3. Detection of Cartilage Defect

 Detection of cartilage defect included two steps. (1) The first step is defining the relationship between the shear wave speed and elastic modulus from a normal cartilage model. The shear deformation and speed were first mapped out by the finite element analysis under the newly determined pushing force within the cartilage. The shear wave speed *C* was determined from distance *d* between two measured sites and the time *t* the wave traveled as

(1) $$\begin{array}{c} C=\frac{d}{t}. \end{array}$$

Based on the literature and our preliminary test, the elastic modulus and shear wave speed might be related to the given *E*, and in a form as

(2) $$\begin{array}{c} C=a\times \sqrt{\frac{E}{2\times \rho \left(1+\mu \right)}}, \end{array}$$

where a was the coefficient to be determined from the finite element simulation [34, 35]. (2) The second step is simulating the cartilage defect. The defect, representing a typical early cartilage lesion [36], had a size of 2 × 2 mm^2^ passing through the entire articular thickness located at the distal site. The predicted elastic modulus was calculated by using (2). The measurement error was determined from the difference between the predicted and given elastic modulus.

## 3. Results

**Figure 1:**
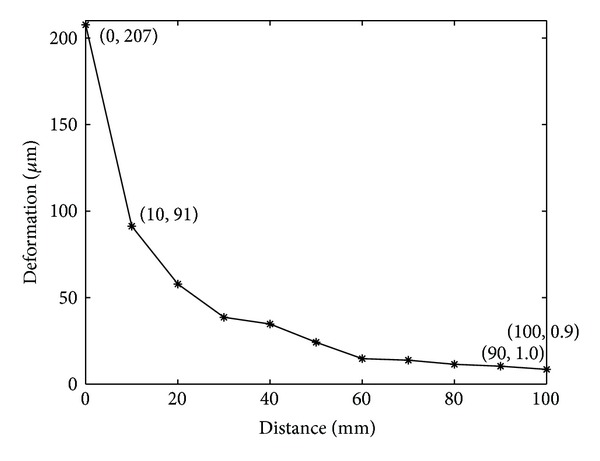
The shear deformation as a function of the distance between the excitation source and the measured site.

 Under the unit pushing force, the shear deformation decreased nonlinearly. The decrease also depended on the duration of the pulse. For a typical duration of 0.5 ms, the deformation declined rapidly during the first 10 mm propagation from 207 *μ*m at 0 mm to 91 *μ*m at 10 mm and slowly decreased to 0.9 *μ*m at the distal site (Figure 1). By meeting the shear deformation at the proximal site and at the distal site with the tested criteria, the pushing force was determined as 10 N.

By using (1), the shear wave speed was calculated as 68.9 m/s. The factor *a* in (2) was then determined as 1.57 and (2) became

(3) $$\begin{array}{c} C=1.57\times \sqrt{\frac{E}{2\times \rho \left(1+\mu \right)}}. \end{array}$$

**Figure 2:**
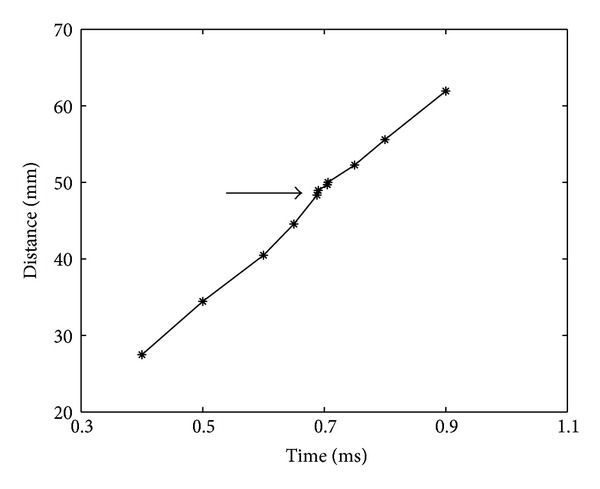
Shear wave front propagation. Perturbation indicated by the arrow was when the wave front passed over the defect site.

**Figure 3:**
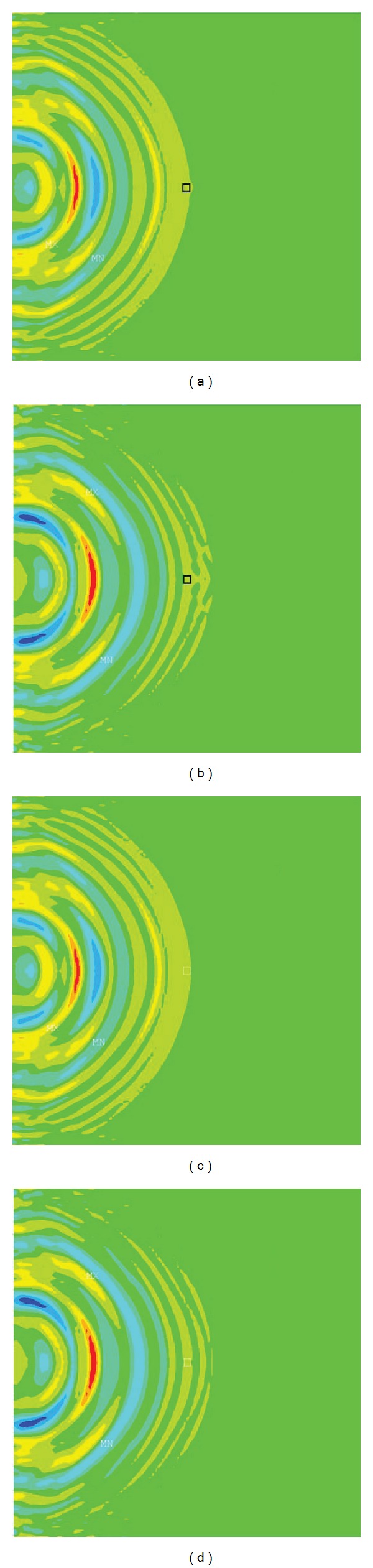
Illustration of shear wave front propagation at the instant (a) when the front reached the defect (black square) and (b) when it passed the defect. (c) and (d) showed the shear wave propagation in the normal cartilage at the two instants, respectively.

**Figure 4:**
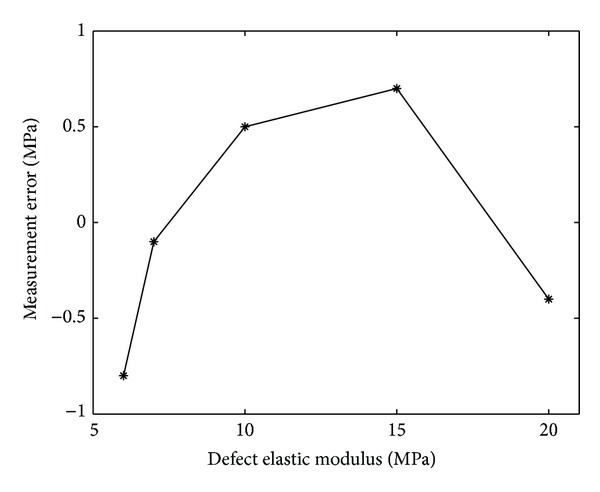
Measurement errors for various elastic moduli of the defect.

The elastography measurement was proportional to the stiffness changes of the defect (Figures 2 and 3). For the simulated defect with an elastic modulus of 7 MPa which was slightly stiffer than the normal cartilage (5 MPa), the measurement error was 0.1 MPa (Figure 4).

## 4. Discussion

 The elastography has been evolved rapidly into the new diagnostic modality. This study proposed a theoretical framework to fulfill the requirement of using the ultrasound elastography to diagnose early OA. The key parameters including the push force magnitude and the measurement accuracy were determined for the guidance of the practical application.

The detection of early OA remains challenging with the current measurement tools. Plain radiography has been commonly used to diagnose OA because it is accessible and relatively inexpensive. Recent techniques with the use of standardized techniques and improved computer algorithms have been shown to be reproducible [37, 38]. However, the plain radiography has a poor sensitivity even for the late stage of OA (66%), keeping it from the diagnosis of early OA. Direct MRI and ultrasound images have not shown to be sensitive in early OA diagnosis [39]. Scintigraphic uptake has been applied to depict specific patterns of OA cartilage, while no pattern has been presented in the early OA [5]. Dual-energy X-ray absorptiometry is a successful tool in the early detection of osteoporosis by measuring bone density. It has been tested in detecting OA in the hip joint [40], but this method has not been able to be used for the early OA detection. Arthroscopy has been used to directly visualize cartilage structure. However, this invasive technique requires specialized skill and is not practical for the early OA diagnosis. Biological markers are the other diagnostic direction which may be achieved through collection of articular synovial fluid. Up to date, markers of inflammation are neither sensitive nor specific enough to monitor the inflammation and damage occurring in the early OA [9, 10].

The theoretical model developed may be oversimplified and can be improved in the future. Firstly, the predicted results should be compared with the experimental measurement to evaluate the accuracy and to guide potential modifications. Secondly, the attenuation of displacement was simulated based mainly on the geometric spreading of the shear wave energy as it propagated outwards from the wave origin, and the wave reflection and refraction were ignored. Although the wave reflection and refraction were important issues, their influence was small because the shear deformation rapidly weakened during the propagation (Figure 1). This issue might become critical when studying smaller joints of wrist and phalanges. Thirdly, the cartilage was assumed as a simplified homogeneous square layer where surface curvature and inhomogeneous were not considered. The location and size variation of the defects were not considered. By more detailed simulation of cartilage subzones and extracellular matrix orientations, a nonuniform and inhomogeneous model can be developed. The cartilage and defect irregularity in geometry should, in theory, affect the ultrasound shear wave propagation. This feature can also be studied in the future with precise 3D reconstructive techniques. Lastly, the surrounding soft tissues from subcutaneous tissues, tendons to muscles may also interfere with the measurement. In practice, the location of the excitation source and receiver will be critical in minimizing the effect. Future simulation of these factors will help address these concerns.

## 5. Conclusions

The theoretical framework was established for detection of early OA based on the noninvasive ultrasound elastography. The pulse pushing force of 10 N was found to be necessary for the excitation, and the relationship between the elastic modulus and shear wave speed was formulated. The results indicated a potential application of the ultrasound elastography to the noninvasive detection of early OA.
